# Aix-La-Chapelle and Its Waters
*"Aix-la-Ohapalle as a Health Resort." English edition by James Donelan, M.B. (London : J. and A. Churchill.)


**Published:** 1892-11-12

**Authors:** 


### Aix-la-Chapelle and its Waters.*

Aix-la-Chapelle has been renowned for its warm mineral waters from immemorial times. The Kelts used them, and the Romans after them, dedicating the springs to Apollo Granius, a deity who was held in high repute as a giver of health. In layer upon layer there lies the testimony of its ancient popularity. Seek beneath the mediæval buildings and you find Roman remains, bricks stamped with the mark of the Sixth Legion, and the remains of *piscina* and *hypocaustum*, with gems and ornaments lost by careless bathers eighteen hundred years ago. Look beneath these again, and you find rougher masonry, which the experts declare to be the workmanship of the aboriginal Kelts. But it is with Charlemagne that the true history of Aachen or Aix-la-Chapelle begins. If the tradition is to be believed in its entirety, the remains of the Roman occupation must have vanished very completely. It is said that while the king was riding through a thicket his horse was frightened by stepping into a hot spring. Charlemagne found out the cause of the animal's alarm, and resolved to build near the spring a palace and a chapel. Other springs were found and surrounded with marble basins, and the fame of Aachen was established. Eginhard, the historian of Charlemagne, speaks of a large basin in which Charlemagne and his suite, numbering sometimes a hundred persons, could bathe and amuse themselves in the water. For it is only in these latter days that people have begun to take their baths as a serious piece of medical treatment; formerly the bathers, robed in all manner of fantastic costumes, spent whole hours in the public bath. Musicians played to them, they received their friends and gossipped with them, played dice or chess on little floating tables, or took the refreshment which the attendant pushed to them on similar tables. It was as good a way of being idle as another; though one may believe that this soaking in hot water all day long was more enervating than health-giving. Charlemagne himself is said to have died from the result of a chill caught after indulging in these prolonged baths.

But the baths proved useful in another, if to the ordinary eye a humbler, way. In the twelfth century a woollen trade arose in the town, and the natural hot springs proved very convenient for the washing of the wool. In 1171, during the construction of the ramparts, a new spring, the Rosen-quelle, was met with. The Rosen-quelle, is now in the "Comphausbad-strasse," and thereby hangs a tale. For "Comphaus," means "Felting-house," and this new warm sulphurous spring was used, not as drink, nor as a bath, but simply to felt the cloth woven in the town. On one occasion the springs threatened to become the reverse of a blessing to the inhabitants. They had by this time built new fortifications, and the Königsbad and Rosen-quelle were within the town. In 1248 the town was besieged by King William of Holland, and he tried, by erecting dams, to prevent the surplus water escaping from the town, and thus to submerge it. By digging a canal the citizens avoided this calamity, but in the end they had to yield to the Dutchman's superior powers, and surrendered after a blockade of six months. The Frieslanders had specially distinguished themselves in the siege, and by way of recompense King William gave them the right of bathing in Aachen free of charge. It is to be hoped that the Frieslanders appreciated the privilege. At this time the baths were crown property, and the city leased them from the king; but by degrees the worthy citizens acquired them, and that just when Dr. Franciscus Blondel was spreading their fame by a book which was translated into various languages, and bringing to them visitors from far and near. Dr. Didier, of Sedan, at that time (1660) Superintendent of the baths, tried to minimise Blondel's services, and between the two medicos arose a very pretty quarrel indeed, conducted largely by pamphleteering, in which Blondel claimed to be the first to introduce the drinking of the waters as a means of cure, although old writings testify that the practice was common long before his day. The need for medical superintendence of the baths is evident when one learns that in the Kaiserbad and the Kleinbad it was necessary for the water to remain from fourteen to eighteen hours before it was sufficiently cool to be safe to enter. What Blondel really deserves credit for—if credit it be—is the amount to which he increased the allowance of water to be drunk. Fourteen glasses a day were thought nothing of, and anything between 200 and 600 ounces was regarded as a reasonable daily allowance for one person. If Mr. Weller, senior, had visited Aix-la-Chapelle in those days he would certainly have seen the visitors "swelling wisibly afore his eyes." It seems that these devoted water-drinkers were rather a mean race, who did not think it necessary to remunerate the "Schenker" who pumped the water into the drinking fountain, and in 1662 an order was given that "the drinkers should give the 'Schenker' something for his trouble, or pump up the water themselves."

If we may believe Blondel, the waters cured everybody of every disease under the sun. Aachen was like the miracle-working shrine that Heine speaks of:—

Thither came many on crutches
Who now dance light on the ground,
And many now play on the fiddle
Who had not a finger sound.

As a matter of fact, however, the ailments which are most successfully treated at Aix-la-Chapelle are gout and rheumatism, with skin diseases and the complaints usually classed with them. The various doctors of Aix-la-Chapelle have united in the writing of the volume now published by Messrs. Churchill, and deal with the ailments which they specially treat. Naturally, while the waters play an important part in all the cures, the same use is not made of them in all diseases. In gout the drinking of the waters is held to be of the first importance, as they help to eliminate the excess of uric acid. The chemical composition of them is such that they seem to absorb and hold in solution an amount of uric acid which otherwise could not be excreted, and would thus set up the disease. Douches and massage are also employed plentifully, and Dr. Mayer, who deals with this disease, is emphatic on the necessity for the "cure" being taken yearly. He quotes many instances in which a patient who had been free from gout during the years he visited the spring, omitted his annual course of treatment and suffered severely during the ensuing twelvemonth. In skin diseases and the like, the baths are supplemented by mercurial inunctions. The method of inunction has been subjected to severe criticism, but in diseases for which mercury is a specific it seems to give the patient all the benefit of the drug; while if due precautions are taken to preserve the organs of respiration from the mercury volatilised by the air, none of the injurious effects of it arise. It must be owned, however, that whatever advantages the inunction of mercury has over its ingestion, the success of the methods employed at Aix-la-Chapelle and other health resorts depends to a considerable extent on the constant medical supervision under which the patient lives. At home his attention to business interferes with his health; at the baths his health is his business. At home he cannot spend the day in rubbing and tubbing, there are too many duties and distractions; and moreover, at home, even though the thermal waters of Aix could be simulated by means of the druggist and kitchen boiler, the appliances and arrangements that have grown up around the spring during the centuries in which it has been in vogue, cannot be obtained in a private house. And lastly, there are the moral helps to health, change of air and scene, a varied company of many nationalities, a new aspect of life. These are not to be ignored among the benefits of Aix-la-Chapelle, and aid the action of the waters discovered by Charlemagne's horse.

* "Aix-la-Chapelle as a Health Resort." English edition by James Donelan, M.B. (London: J. and A. Churchill.)

